# Clindamycin resistance among *Staphylococcus aureus* causing skin and ear infections from Chennai, South India

**DOI:** 10.1186/1471-2334-12-S1-P70

**Published:** 2012-05-04

**Authors:** Betsy S Dass, A Nagarajan, Padma Krishnan, G Sivakumar

**Affiliations:** 1 Dept. of Microbiology, Dr. ALM PGIBMS, University of Madras, Chennai, India; 2 Dept. of General Surgery, Rajiv Gandhi Govt. General Hospital, Chennai, India

## Background

Antibiotic resistance in *S. aureus* is one of the major concerns and the rate of MRSA has dramatically increased in recent years. Clindamycin belongs to the MLS group and is used to treat skin and soft tissue infections. Resistance to clindamycin may be inducible (iMLSB) or constitutive (cMLSB) and it is present both in MRSA and MSSA. Treatment failure has been reported both in iMLSB and cMLSB cases. Knowledge on clindamycin resistance is important for its proper use. Hence, the present study was done to detect clindamycin resistance among *S. aureus* causing skin and ear infections.

## Methods

84 samples (skin – 55, ear discharge – 29) were collected from the OPD and wards of tertiary hospital in Chennai. Isolation and identification of *S. aureus* was done according to standard protocol. Antibiotic susceptibility pattern was tested for all isolates to various antibiotics. MRSA screening was done by cefoxitin disc diffusion method. Erythromycin induced clindamycin resistance was detected by using D-test. MIC was determined by agar dilution method.

## Results

From the 84 clinical samples, 49 *S. aureus* isolates were obtained of which 32 (65%) were MRSA. 32 (65%) strains were resistant to erythromycin and 3 (6.1%) showed resistance to clindamycin with MIC of 256μg/ml. Of the 32 erythromycin resistant *S. aureus* isolates, 18 exhibited inducible clindamycin resistance. All clindamycin resistant isolates were found to be MRSA.

## Conclusion

Increasing resistance to clindamycin has been found among MRSA indicating urgent need for guidelines on its proper use to decrease the morbidity and mortality by MRSA infections.

